# Multilayered regulation of iron homeostasis in Arabidopsis

**DOI:** 10.3389/fpls.2023.1250588

**Published:** 2023-09-29

**Authors:** Julien Spielmann, Steven Fanara, Valérie Cotelle, Grégory Vert

**Affiliations:** ^1^ Plant Science Research Laboratory (LRSV), University of Toulouse, CNRS, UPS, Toulouse INP, Auzeville-Tolosane, France; ^2^ InBioS-PhytoSystems, Functional Genomics and Plant Molecular Imaging, Department of Life Sciences, University of Liège, Liège, Belgium

**Keywords:** iron, Fe, iron homeostasis, iron uptake, transcriptional and post-translational regulations, transcription factors, iron transporters, intracellular trafficking

## Abstract

Iron (Fe) is an essential micronutrient for plant growth and development due to its role in crucial processes such as photosynthesis and modulation of the redox state as an electron donor. While Fe is one of the five most abundant metals in the Earth’s crust, it is poorly accessible to plants in alkaline soils due to the formation of insoluble complexes. To limit Fe deficiency symptoms, plant have developed a highly sophisticated regulation network including Fe sensing, transcriptional regulation of Fe-deficiency responsive genes, and post-translational modifications of Fe transporters. In this mini-review, we detail how plants perceive intracellular Fe status and how they regulate transporters involved in Fe uptake through a complex cascade of transcription factors. We also describe the current knowledge about intracellular trafficking, including secretion to the plasma membrane, endocytosis, recycling, and degradation of the two main Fe transporters, IRON-REGULATED TRANSPORTER 1 (IRT1) and NATURAL RESISTANCE ASSOCIATED MACROPHAGE PROTEIN 1 (NRAMP1). Regulation of these transporters by their non-Fe substrates is discussed in relation to their functional role to avoid accumulation of these toxic metals during Fe limitation.

## 1 Introduction

Iron (Fe) is a crucial micronutrient for most living organism due to its redox properties. Thanks to its ability to easily transfer electrons (gain or lose), Fe serves as co-factor in many vital chemical reactions such as cellular respiration, DNA synthesis and photosynthesis (Hänsch and Mendel, 2009; Palmer and Guerinot, 2009; Rengel et al., 2023). Consequently, perturbation of Fe homeostasis leads to several growth, developmental or health symptoms (Briat et al., 1995). According to the World Health Organization, Fe deficiency in humans is the most severe nutritional issue in the world, with anemia caused by dietary Fe deficiency affecting around 15% of the world’s population (World Health Organization, 2016; Safiri et al., 2021). In plants, Fe starvation is associated with both reduced growth and chlorosis (Briat et al., 1995). Although Fe is the second most abundant metal in the Earth’s crust, its poor solubility renders Fe limiting for plant growth (Rengel et al., 2023). In particular, alkaline and aerobic soil conditions favor Fe oxidation and precipitation of insoluble ferric (Fe^3+^) complexes. To counteract the Fe unavailability, plants have developed two different Fe acquisition strategies.

The first one, referred to as strategy I, is a reduction-based strategy used by all plant species except grasses to absorb Fe. It consists in a multistep process depending on a local rhizosphere acidification by root-released protons, a subsequent Fe reduction (Fe^3+^ to Fe^2+^) and the ferrous iron (Fe^2+^) uptake into root cells (Briat et al., 1995; Schmidt, 2003; Briat et al., 2015). In the model plant *Arabidopsis thaliana*, strategy I is driven by a complex of three proteins localized to the plasma membrane of root epidermal cells. First, the ARABIDOPSIS H^+^-ATPase 2 (AHA2) proton pump actively exports protons into the rhizosphere to reduce the pH, hence increasing Fe^3+^ solubility. Then, the FERRIC REDUCTION OXIDASE 2 (FRO2) reduces ferric iron into Fe^2+^ at the root surface. Finally, the high-affinity Fe transporter IRON-REGULATED TRANSPORTER 1 (IRT1) transports Fe^2+^ into root epidermal cells (Robinson et al., 1999; Vert et al., 2002; Santi and Schmidt, 2009). Upon Fe starvation, IRT1 is the major Fe transporter. In Fe-replete conditions however, the metal transporter NATURAL RESISTANCE-ASSOCIATED MACROPHAGE PROTEIN 1 (NRAMP1) cooperates with IRT1 and takes part in the Fe^2+^ uptake process as a low-affinity transporter (Castaings et al., 2016). Plants using strategy I also secrete secondary metabolites such as coumarins through the ATP-binding cassette (ABC) transporter ABCG37 (also named PLEIOTROPIC DRUG RESISTANCE 9: PDR9) to increase Fe mobility in the rhizosphere (Fourcroy et al., 2014). As coumarins play an important role in alkaline soils, it has been hypothesized that they might also form complexes with Fe^3+^ that could be directly taken up into the root to optimize Fe uptake (Robe et al., 2021). Graminaceous plants use a chelation-based strategy for Fe acquisition, named strategy II. They massively excrete phytosiderophores such as mugineic acids (MAs) (Takagi et al., 1984; Schmidt, 2003). MAs are secreted in the root vicinity through the TRANSPORTER OF MUGINEIC ACID 1 (TOM1) efflux transporter, to chelate and solubilize Fe^3+^ (Takagi et al., 1984; Nozoye et al., 2011; Nozoye et al., 2015). Fe-MA complexes are then taken up into root cells by the YELLOW STRIPE 1 (YS1)/YS1-LIKE (YSL) transporter (Inoue et al., 2009; Lee et al., 2009).

Mechanisms involved in strategy II will not be further discussed in this mini-review which focusses on the regulation of Fe uptake processes dedicated to strategy I. First, we will give a brief overview of Fe status sensing and the following steps regulating Fe homeostasis in plants, including the rapid transcriptional and post-transcriptional events occurring upon Fe deficiency. In a second part, we will summarize the current knowledge on the intracellular trafficking and post-translational regulation of the two main Arabidopsis Fe^2+^ transporters.

## 2 Gene regulation upon Fe deficiency

### 2.1 Transcriptional regulation

**Figure 1:**
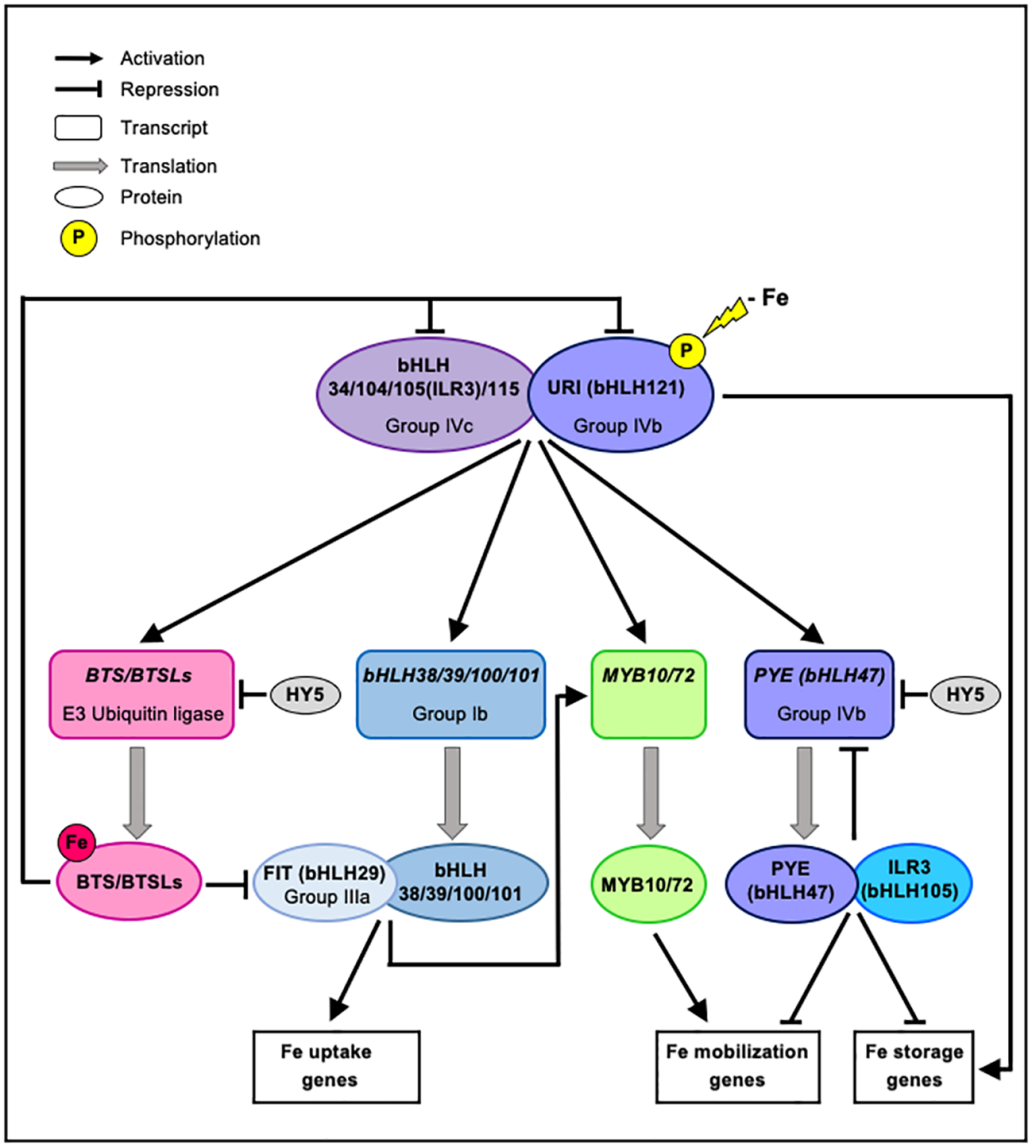
Model for transcriptional regulation of Fe homeostasis. Upon low-Fe conditions, URI (bHLH121) is phosphorylated and interacts with subgroup IVc bHLH transcription factors (bHLH34/104/105(ILR3)/115). These heterodimers activate the expression of several genes involved in the regulation of Fe homeostasis, including genes encoding the E3 ubiquitin ligases BTS/BTSLs, and the MYB10, MYB72, PYE (bHLH47) and subgroup Ib bHLH (bHLH38/39/100/101) transcription factors. In contrast, the bZIP transcription factor HY5 negatively regulate the expression of *BTS* and *PYE*. Members of the bHLH Ib subgroup form heterodimers with FIT (bHLH29) to promote the expression of *MYB10/72* and of genes involved in Fe uptake. PYE (bHLH47), a subgroup IVb bHLH, interacts with ILR3 (bHLH105) to negatively regulates its own expression and also to act as a transcriptional repressor of genes involved in Fe storage which are otherwise induced by URI (bHLH121). PYE (bHLH47) with ILR3 (bHLH105) likely represses the expression of genes involved in Fe mobilization that are induced under the control of MYB10/72. PYE also negatively regulates the expression of bHLH Ib genes (not shown). When Fe becomes available, E3 ubiquitin ligases of the BTS/BTSLs family target bHLH transcription factors (bHLH29(FIT)/104/105(ILR3)/115/121(URI)) leading to their degradation via the 26S proteasome to turn off Fe-deficiency signaling and prevent Fe overload. For clarity, the involvement of IMA peptides in the transcriptional cascade controlling Fe homeostasis is not shown, but is discussed in the text.

Fe homeostasis is controlled by a transcriptional cascade involving an intricate network of transcription factors (TFs) regulating the expression of genes encoding proteins involved in Fe uptake, distribution and storage (**Figure 1**). Fe depletion at the vicinity of plant roots induces the phosphorylation of the basic helix-loop-helix (bHLH) transcription factor bHLH121/UPSTREAM REGULATOR OF IRT1 (URI) and its root cellular relocalization from the central cylinder and the endodermis to the cortex and the epidermis (Gao et al., 2020a). Phosphorylated URI then forms heterodimers with members of the bHLH subgroup IVc (bHLH34, bHLH104, bHLH105/IAA-LEUCINE RESISTANT 3 (ILR3) and bHLH115) (Zhang et al., 2015; Li et al., 2016b; Liang et al., 2017; Kim et al., 2019; Tissot et al., 2019; Gao et al., 2020a; Lei et al., 2020). Cooperatively, these TFs acts as direct transcriptional activators of key genes involved in the Fe regulatory network, including four bHLH Ib genes (*bHLH38, bHLH39, bHLH100, bHLH101*), *POPEYE* (*PYE*/*bHLH47*), as well as *BRUTUS* (*BTS*) and *BRUTUS LIKE 1* (BTSL1), which both encode E3 ubiquitin ligases (Kim et al., 2019; Gao et al., 2020a; Lei et al., 2020). In turn, members of the bHLH Ib subgroup associate with bHLH29/FER-LIKE IRON DEFICIENCY INDUCED TRANSCRIPTION FACTOR (FIT) to form heterodimers that promote the expression of the Fe uptake machinery (Colangelo and Guerinot, 2004; Jakoby et al., 2004; Yuan et al., 2008). PYE, a bHLH of the subgroup IVb, interacts with bHLH105/ILR3, to act as a transcriptional repressor of its own expression and of the expression of *FERRITIN* (*FER*) genes involved in Fe storage (Long et al., 2010; Samira et al., 2018; Tissot et al., 2019; Akmakjian et al., 2021), which are otherwise induced by bHLH121/URI (Gao et al., 2020b). PYE, likely in interaction with ILR3, also controls Fe redistribution, especially regarding its mobility, through the negative control imposed on the expression of *NAS4* (*NICOTIANAMINE SYNTHASE 4*) involved in the synthesis of nicotianamine (NA), a Fe chelator important for intercellular Fe transport, and on the expression of *ZINC-INDUCED FACILITATOR 1* (*ZIF1*) involved in the vacuolar sequestration of NA in roots (Long et al., 2010; Haydon et al., 2012; Schuler et al., 2012; Tissot et al., 2019). PYE was also found to repress the transcription of bHLH Ib genes (bHLH38, bHLH39, bHLH100, and bHLH101) by directly binding to their promoters, hence triggering the subsequent downregulation of *IRT1* and *FRO2* (Pu and Liang, 2023). Moreover, two transcription factors of the myeloblastosis (MYB) family, MYB10 and MYB72, cooperate to positively regulate *NAS2* and *NAS4* expression (Palmer et al., 2013), and the expression of both *MYB* is induced upon Fe deficiency under the control of heterodimeric complexes FIT/bHLH Ib and might also be dependent on bHLH121/URI (Palmer et al., 2013; Gao et al., 2020a; Lei et al., 2020).

Fine tuning of this complex transcriptional regulatory cascade may be achieved by the Fe-deficiency-dependent phosphorylation of bHLH121/URI and its subsequent phospho-dependent degradation by BTS (Kim et al., 2019). It is noteworthy, however, that *BTS* expression pattern is restrained to the stele (Selote et al., 2015; Rodríguez-Celma et al., 2019), where BTS would coexist with an unphosphorylated, hence undegradable bHLH121/URI protein (Kim et al., 2019). Furthermore, no interaction was uncovered between bHLH121/URI and BTS or BTSL proteins in yeast two-hybrid assays (Gao et al., 2020a). BTS and its homologs BTSL1 and BTSL2 are all E3 ubiquitin ligases that are able to bind Fe and to adjust their biological function accordingly to negatively regulate Fe homeostasis (Selote et al., 2015; Hindt et al., 2017; Kim et al., 2019). The negative control imposed by BTS and BTSLs results from the proteasomal degradation of their targets, among which are found several bHLHs especially important for Fe homeostasis (e.g. bHLH29/FIT, bHLH104, bHLH105/ILR3 and bHLH115) (Selote et al., 2015; Rodríguez-Celma et al., 2019). BTS and BTSL proteins all possess hemerythrin/HHE Fe-binding domains at their N-terminus (Hindt et al., 2017), and the BTS protein is stabilized in the absence of Fe bound to glutamic acid residues located within HHE domains (Selote et al., 2015). At the transcriptional level, *BTS* and *BTSL* genes are upregulated by Fe deficiency at least through the control of three bHLH transcription factors (bHLH105/ILR3, bHLH115 and bHLH121/URI) (Long et al., 2010; Liang et al., 2017; Rodríguez-Celma et al., 2019; Gao et al., 2020a). In contrast, ELONGATED HYPOCOTYL 5 (HY5), a light signaling basic leucine zipper (bZIP) transcription factor, has been identified as a negative regulator of *BTS* and *PYE* expression to modulate Fe-deficiency responses (Mankotia et al., 2023). It was recently shown that IRON MAN (IMA) peptides interact with and are ubiquitinated by BTS, which inhibits the degradation of bHLH105 and bHLH115 through binding competition (Li et al., 2021). In the same line of evidence, the interaction of BTSL1 with PYE and bHLHs of the subgroup IVc is attenuated by the peptide IMA1 (Lichtblau et al., 2022). Interestingly, mutation applied to the *bHLH121/URI* gene leads to an up-regulation of *IMA1/2/3* and *BTS* genes under Fe deficiency (Lei et al., 2020).

Interestingly, crosstalks exist between Fe homeostasis and phytohormone signaling pathways. Indeed, FIT interacts with ETHYLENE INSENSITIVE 3 (EIN3) and ETHYLENE INSENSITIVE 3-LIKE 1 (EIL1) (Lingam et al., 2011). Two components of the jasmonic acid (JA) pathway, MYC2 and JAR1, negatively regulate the expression of *FIT* and *bHLH Ib* genes in response to JA (Cui et al., 2018). Moreover, FIT interactions with four IVa bHLHs (bHLH18, bHLH19, bHLH20 and bHLH25) trigger JA-mediated FIT degradation by the 26S proteasome (Cui et al., 2018). *FIT*, *bHLH38* and *bHLH39*, as well as *FRO2*, *IRT1*, expression is also positively regulated by two key enzymes of the gibberellin (GA) biosynthetic pathway (gibberellin 3-oxidase 1 and 2, GA3ox1 and GA3ox2) (Matsuoka et al., 2014). DELLA proteins, which are negatively regulated by bioactive GA, associate with FIT, bHLH38 and bHLH39 to inhibit their activity (Wild et al., 2016).

*FIT* and Ib bHLH genes, hence their direct targets (at least *IRT1* (Schwarz and Bauer, 2020) and putatively *NRAMP1*, as they are known to be co-regulated (Colangelo and Guerinot, 2004)) are positively regulated by a wide range of metal excess (zinc (Zn^2+^), cobalt (Co^2+^), nickel (Ni^2+^), cadmium (Cd^2+^) and manganese (Mn^2+^)) (Wu et al., 2012; Lešková et al., 2017). The effect of metal excess on the expression of aforementioned genes likely results from competition with Fe uptake as it was demonstrated for cadmium (Wu et al., 2012).

### 2.2 Post-transcriptional regulation

Upon Fe deficiency, many genes mentioned above undergo changes not only at the transcriptional level but also at the post-transcriptional level through modification of their splicing patterns (Lan et al., 2013; Li et al., 2013; Li et al., 2016a; Hsieh et al., 2022). Alternative splicing events occurring upon Fe deficiency might be regulated by many splicing factors, including members of the serine/arginine-rich (SR) family (Zhang et al., 2014; Dong et al., 2018; Fanara et al., 2022). Although an alternatively spliced isoform of *IRT1* mRNA retaining an intron has been previously described (Li et al., 2013), no significant changes appear in the *IRT1* splicing profile in a wild-type plant upon long- or short-term Fe deficiency (Fanara et al., 2022; Hsieh et al., 2022). Among the genes of the bHLH Ib subgroup, only *bHLH100* and *bHLH101* undergo differential intron retention (DIR) upon short-term Fe deficiency (Hsieh et al., 2022). While *bHLH29/FIT* intensively undergoes differential donor or acceptor (DDA) splice sites events (Hsieh et al., 2022), changes in the *NRAMP1* splicing profile occur through both DIR (Lan et al., 2013; Li et al., 2013) and DDA (Lan et al., 2013; Li et al., 2016a). DDA events define new borders between exon and intron at the 5’ and 3’ ends of the intron, respectively, and might result in frameshifts and subsequent premature termination codons. Alternatively, if in-frame, DDA could modify the length of exonic regions (English et al., 2010). Alternative splicing therefore has the potential to partially modify important domain of a protein, hence modifying its interaction network, function, stability, or even its intracellular localization (English et al., 2010; Kalyna et al., 2012; Drechsel et al., 2013; Remy et al., 2013).

## 3 Intracellular trafficking and post-translational regulation of Fe transporters

As previously mentioned, Fe^2+^ is taken up by the cooperation of two transporters, NRAMP1 and IRT1 in plants (Curie et al., 2000; Vert et al., 2002; Castaings et al., 2016). The low affinity Fe transporter NRAMP1 plays an important role in Fe-replete conditions to support Fe homeostasis. However, upon Fe deficiency, the high affinity Fe transporter IRT1 is rapidly and strongly induced to ensure efficient Fe uptake.

### 3.1 Secretion of NRAMP1 and IRT1 to the plasma membrane

Membrane proteins are synthesized in the endoplasmic reticulum (ER) (High and Laird, 1997), and use well-characterized secretion signals and pathways to traffic in the cell to their destination (Peer, 2011; Chung et al., 2016). Exit from the ER requires the direct interaction with the coat protein complex II (COPII) using specific motifs, which allows their transport to the Golgi apparatus (Barlowe et al., 1994; Matheson et al., 2006; Lord et al., 2013). Then, membrane proteins follow the secretory pathway through the *trans*-Golgi network (TGN) and reach their final destination using various vesicles such as clathrin-coated vesicles or exocysts (Matheson et al., 2006; Peer, 2011; Chung et al., 2016). The mechanisms by which IRT1 and NRAMP1 reach the plasma membrane is still elusive and lacks experimental evidence. However, one of the first characterized ER export motifs, the diacidic motif (D/E)X(D/E), is present in both transporters (Hanton et al., 2005). NRAMP1 harbors a diacidic motif (DVD) in its cytoplasmic C-terminal part, as many others membrane proteins, and IRT1 possesses the diacidic motif (EDD) in its largest cytoplasmic loop. Besides, mutation of this motif in IRT1 from *Malus xiaojinesis* leads to its retention into the ER (Tan et al., 2018). In addition, two of the five COPII core components, SEC13a and SEC31b, were recently identified in the *Arabidopsis* IRT1 interactome (Matheson et al., 2006; Lord et al., 2013; Martìn-Barranco et al., 2020). Altogether, these findings suggest that IRT1 is exported from the ER to the Golgi apparatus using the COPII machinery. Then, during the post-Golgi trafficking, the CHOLINE TRANSPORTER-LIKE 1 (CTL1) plays an essential role to allow the delivery of NRAMP1 and probably IRT1 at the plasma membrane (Gao et al., 2017).

### 3.2 Post-translational regulation of NRAMP1 by non-Fe metals

**Figure 2:**
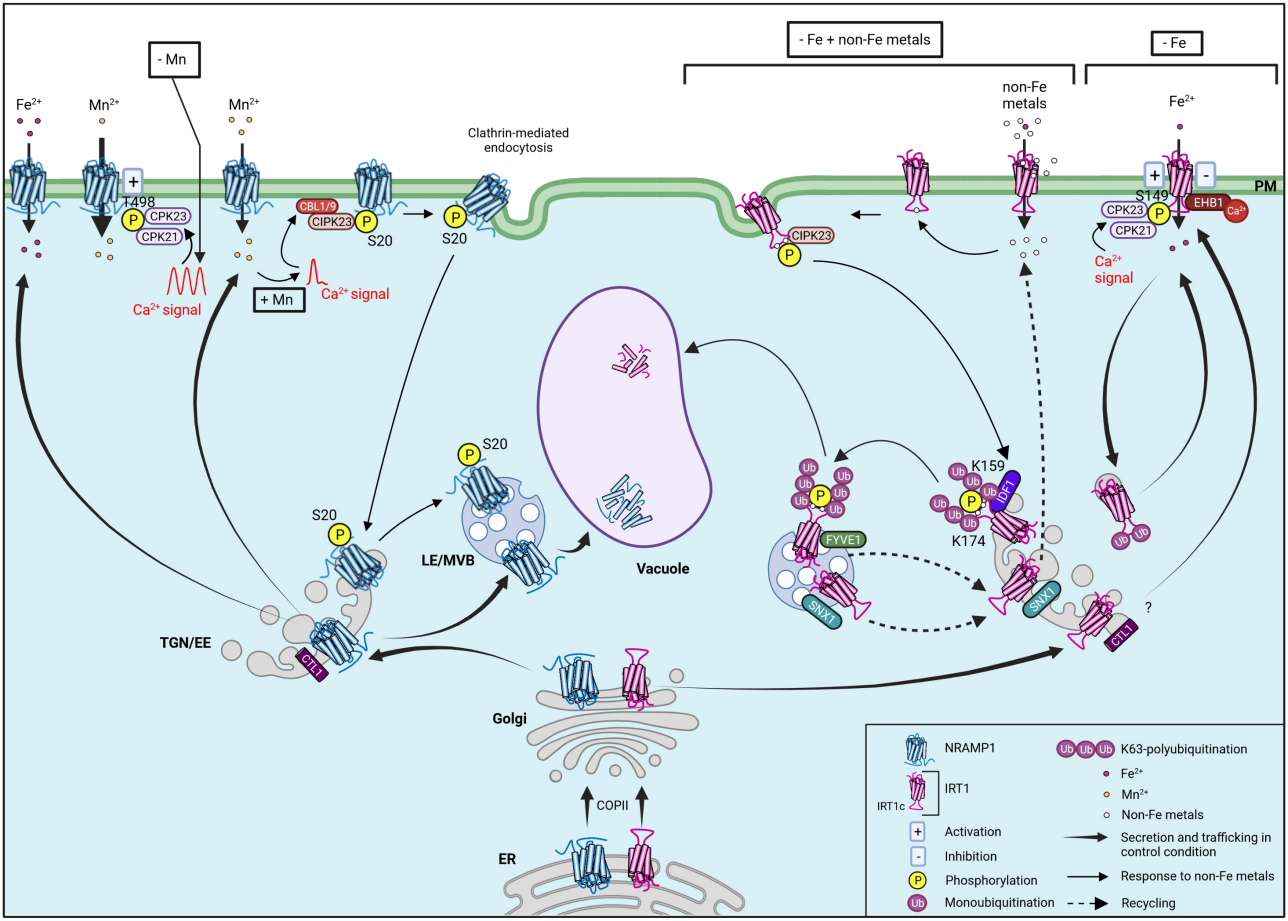
Model summarizing intracellular trafficking and post-translational regulation of NRAMP1 and IRT1. The two Fe transporters NRAMP1 and IRT1 are synthesized in the ER and exit this compartment by interacting with COPII. Then, the proteins follow the secretory pathway through the EE/TGN and reach the PM by a process involving CTL1 for NRAMP1 and probably also for IRT1. At the PM, the low affinity Fe^2+^ transporter NRAMP1 plays an important role in Fe-replete conditions, whereas the high affinity Fe^2+^ transporter IRT1 is induced upon Fe deficiency to ensure efficient Fe uptake. In control condition, NRAMP1 is found at the PM, the TGN/EE, the LE/MVB and also undergoes vacuolar degradation. NRAMP1 steady-state localization pattern is dependent on endocytosis (not shown). Besides Fe^2+^ transport, NRAMP1 is also essential for Mn^2+^ uptake. Mn^2+^ depletion (- Mn) induces intracellular Ca^2+^ oscillations decoded by CPK21 and CPK23 which phosphorylate NRAMP1 at T498 leading to NRAMP1 activation and increased Mn^2+^ uptake. The increase in intracellular Mn^2+^ concentration (+ Mn) induces a transient Ca^2+^ signal received by CBL1 and CBL9 located on the PM which activate CIPK23 to interact with and phosphorylate NRAMP1 at S20. This phosphorylation induces clathrin-mediated endocytosis of NRAMP1 reducing NRAMP1 distribution on the PM while enhancing its presence in the endosomal fraction to prevent Mn^2+^ toxicity. In addition to Fe, IRT1 also transports non-Fe metals (Zn^2+^, Mn^2+^, Co^2+^, Cd^2+^). In the combined absence of Fe and non-Fe metals, IRT1 is localized in the outer PM domain facing the rhizosphere in root epidermal cells. When Fe is limited and non Fe-metals are present at physiological concentration (- Fe), IRT1 is multimonoubiquinated and cycles between the PM and TGN/EE. In these conditions, the Fe transport activity of IRT1 is negatively regulated by Ca^2+^-promoted interaction of EHB1 with IRT1. In contrast, the phosphorylation of S149 in IRT1c by Ca^2+^-activated CPK21 and CPK23 stimulates IRT1 transport activity to promote Fe uptake. In Fe-depleted and non-Fe metal excess conditions (- Fe + non-Fe metals), IRT1 is removed from the PM. First, the high influx rate of non-Fe metals is directly sensed by IRT1 through non-Fe metal binding to histidine residues within IRT1c. This interaction triggers the recruitment of CIPK23 which phosphorylates S/T residues within IRT1c, creating a docking site for the E3 ubiquitin ligase IDF1, which decorates K159 and K174 in IRT1c with K63-linked polyubiquitin chains. IRT1 is then sorted toward LE/MVB for subsequent degradation in the vacuole. IRT1 may be recycled to earlier endocytic compartments or the PM through pathways involving SNX1 and FYVE1. IRT1 is also thought to be degraded via the 26S proteasome and autophagy pathways in response to Cd^2+^ stress (not shown). To simplify the scheme, only key proteins are shown (SEC13a, SEC31b, PH1, UBC35, UBC36, ESCRT complex, ATL31 and PATL2 are not represented). ER endoplasmic reticulum, TGN/EE *trans*-Golgi network/early endosome, LE/MVB late endosome/multivesicular body.

Interestingly, both transporters have low Fe specificity allowing the entry, in plant cells, of others divalent metals (hereafter named non-Fe metals), such as Zn^2+^, Mn^2+^, Co^2+^, and Cd^2+^ (Vert et al., 2002; Cailliatte et al., 2010; Barberon et al., 2011). In order to fine tune Fe acquisition and hence limit toxicity of their non-Fe metal substrates, both transporters are regulated at the post-translational level leading to a complex intracellular trafficking (**Figure 2**) (Barberon et al., 2011; Barberon et al., 2014; Agorio et al., 2017; Dubeaux et al., 2018; Castaings et al., 2021; Spielmann et al., 2022). Besides Fe transport, NRAMP1 is also essential for manganese uptake (Curie et al., 2000; Cailliatte et al., 2010; Castaings et al., 2016). Indeed, manganese availability impacts NRAMP1 subcellular localization. In control condition, NRAMP1 is shared between plasma membrane and endosomal vesicles of the *trans*-Golgi network/early endosome (TGN/EE) and the late endosome/multivesicular body (LE/MVB) (Agorio et al., 2017; Castaings et al., 2021). Increasing manganese availability gradually reduces the amount of NRAMP1 at the plasma membrane while enhancing its presence in the endosomal fraction (Castaings et al., 2021). This manganese-induced NRAMP1 endocytosis involves the clathrin-mediated endocytic pathway and occurs after phosphorylation of NRAMP1 (Castaings et al., 2021). The increase of intracellular manganese concentration seems to induce an intracellular transient calcium signal which is perceived by two calcium sensors, the CALCINEURIN-B-LIKE 1 (CBL1) and CALCINEURIN-B-LIKE 9 (CBL9). CBL1/9 interact with and activate the CBL-INTERACTING PROTEIN KINASE 23 (CIPK23) which phosphorylates NRAMP1 at serine 20 (S20), thereby inducing endocytosis of NRAMP1 to reduce its distribution on the plasma membrane and enhance plant tolerance to manganese toxicity. Indeed, in *cipk23* and *cbl1/cbl9* mutant backgrounds, NRAMP1 manganese-mediated endocytosis is abolished (Zhang et al., 2023b). Additionally, a nonphosphorylatable NRAMP1 mutant on S20 is blocked at the plasma membrane unlike the corresponding phosphomimic variant which is constitutively endocytosed, thus suggesting that phosphorylation is a pivotal step for NRAMP1 trafficking (Castaings et al., 2021). Finally, NRAMP1 undergoes vacuolar degradation through an unclear process depending on the PLECKSTRIN HOMOLOGY (PH) DOMAIN-CONTAINING PROTEIN 1 (PH1), but independent of manganese availability (Agorio et al., 2017; Castaings et al., 2021). Even if perturbation of NRAMP1 endocytosis was found to cause manganese toxicity (Castaings et al., 2021), most factors and mechanisms driving NRAMP1 trafficking and degradation remain to be identified and characterized.

### 3.3 Post-translational regulation of IRT1 by non-Fe metals

In the absence of Fe and non-Fe metals, IRT1 is mainly localized at the plasma membrane of root epidermal cells in a soil-facing polar fashion to optimize Fe uptake (Barberon et al., 2014; Dubeaux et al., 2018). When plants are exposed to non-Fe metals, IRT1 is subjected to a rapid and complex post-translational regulation to limit accumulation and toxicity of non-Fe metals. IRT1 acts as a transceptor, combining transporter and receptor activities, enabling sensing of intracellular metal status and regulation of its cell surface levels (Cointry and Vert, 2019). Increasing the concentration of non-Fe metals gradually removes IRT1 from the cell surface, allowing IRT1 to traffic through endosomal compartments on its way to vacuolar degradation (Dubeaux et al., 2018). Thanks to a histidine-rich motif located in the largest cytosolic loop (thereafter called IRT1c), IRT1 directly binds non-Fe metals (Dubeaux et al., 2018; Spielmann et al., 2022). This interaction triggers the recruitment of the CIPK23 kinase that phosphorylates IRT1c and thus creates a docking site for the E3 ubiquitin ligase IRON DEGRADATION FACTOR 1 (IDF1) (Shin et al., 2013; Dubeaux et al., 2018). Through the cooperation with two E2 UBIQUITIN CONJUGATING ENZYMES 35 and 36 (UBC35 and UBC36), IDF1 decorates two IRT1c lysine residues (K159 and K174) with K63-linked polyubiquitin chains (Shin et al., 2013; Dubeaux et al., 2018; Spielmann et al., 2022). IRT1 is then sorted toward late endosomes (LEs) and the vacuole for degradation, but the exact underlying mechanisms remain unclear (Barberon et al., 2011; Dubeaux et al., 2018; Spielmann et al., 2022). Polyubiquitinated IRT1 is probably recognized by the ENDOSOMAL SORTING COMPLEX REQUIRED FOR TRANSPORT (ESCRT) complex to be sent to the vacuole for degradation unless recycled (Dubeaux and Vert, 2017). In fact, two recycling mechanisms have been identified to exit the route to the vacuole (Barberon et al., 2014; Ivanov et al., 2014). One of these pathway preventing IRT1 degradation involves the SORTING NEXIN 1 (SNX1) protein which partly co-localise with IRT1 in TGN/EE and to a lesser extent in LE. A *snx1* loss-of-function plant displays lower IRT1 levels than wild-type plants due to enhanced degradation rate. This suggests a role for SNX1 in the recycling of IRT1 (Ivanov et al., 2014). Secondly, IRT1 interacts with the phosphatidylinositol-3-phosphate-binding protein FYVE1 in LE, which alters its fate. Indeed, plants with enhanced expression of FYVE1 not only show an accumulation of IRT1 at the plasma membrane, but also display a loss of IRT1 lateral polarity (Barberon et al., 2014). Considering that FYVE1 is part of the ESCRT complex and mediates MVB sorting (Gao et al., 2014), the mechanisms by which FYVE1 overexpression reroutes IRT1 to the plasma membrane are still unclear.

IRT1 was also recently demonstrated to be degraded via the 26S proteasome and autophagy pathways. Indeed, IRT1 directly interacts with the plasma membrane-localized RING-type E3 ubiquitin ligase ARABIDOPSIS TOXICOS EN LEVADURA 31 (ATL31), which enhances IRT1 ubiquitination and leads to both proteasome- and autophagy-dependent degradation in response to cadmium stress. In addition, the WRKY33 transcription factor directly activates *ATL31* expression in response to cadmium stress, suggesting a WRKY33-ATL31-IRT1 regulatory module involved in cadmium-specific plant tolerance (Zhang et al., 2023a). It remains to be determined how the proteasome may convey the degradation of a highly hydrophobic membrane protein like IRT1.

Finally, IRT1 post-translational regulatory mechanisms have been proposed to modulate Fe uptake. The peripheral membrane protein ENHANCED BENDING 1 (EHB1), a calcium-binding protein, acts as an Fe uptake inhibitor even during Fe starvation. EHB1 binds to IRT1c and thus interacts with IRT1 in a calcium-dependent manner, reducing Fe acquisition by a still unknown process. The IRT1-EHB1 interaction might represent a rapid mechanism to switch off Fe import once its optimal intracellular concentration has been reached, avoiding Fe overaccumulation and toxicity (Khan et al., 2019). The peripheral plasma membrane SEC14-Golgi dynamics (SEC14-GOLD) protein PATELLIN 2 (PATL2) also binds IRT1c to reduce membrane oxidative damage during Fe import via IRT1 (Hornbergs et al., 2023). In contrast, Fe deficiency leads to phosphorylation of serine 149 (S149) in IRT1c by the CALCIUM-DEPENDENT PROTEIN KINASE 21 and 23 (CPK21 and CPK23) calcium-regulated kinases to increase IRT1 transport activity to optimize Fe uptake (Wang et al., 2023). In response to manganese depletion, these two kinases also catalyze the phosphorylation of NRAMP1 at threonine 498 (T498), leading to NRAMP1 activation and optimal manganese uptake (Fu et al., 2022).

## 4 Conclusions and future perspectives

The control of cellular Fe relies on a complex pathway regulated at several levels and highly integrated. Fe deficiency quickly leads to an extensive cascade of transcriptional regulation mainly controlled by key members of the bHLH family and Fe-dependent protein degradation governed by BTS/BTSLs. Post-transcriptional events, including splicing, add a new level of complexity leading to a plethora of mRNAs rapidly responding to Fe deficiency and creating a diverse proteome controlling Fe uptake, as well as its intra- and intercellular Fe distribution. The trafficking and post-translational regulation of both NRAMP1 and IRT1 emerge as crucial for proper metal uptake and reveal some interesting parallels between the two transporters. Indeed, many post-translational modifications may affect the activity of these transporters, such as the phosphorylation of critical residues leading to their activation in limiting conditions or to their removal from the plasma membrane to avoid toxicity under metal stress. Finally, the polarity of membrane transporters is also inherently connected to their ability to drive directional Fe and metal uptake. Mechanisms controlling polar localization and maintenance of transporters therefore deserve more attention.

## Author contributions

All authors listed have made a substantial, direct, and intellectual contribution to the work and approved it for publication.
